# Public awareness, knowledge gaps, and health anxiety concerning microplastics in human blood: a cross-sectional survey of Indian adults

**DOI:** 10.3389/fpubh.2026.1786204

**Published:** 2026-03-11

**Authors:** Abdullah M. AlShahrani, Anupriya Kumari, Ajay Kumar Behera, S. Rehan Ahmad

**Affiliations:** 1 Department of Basic Medical Science, College of Applied Medical Sciences, King Khalid University (KKU), Abha, Saudi Arabia; 2 Shri Sant Tukaram Shikshan Prasarak Mandal’s Adhyapak Mahavidyalaya, Savitribai Phule Pune University, Pune, India; 3 Indira Gandhi National Open University (IGNOU), Regional Center, Kolkata, West Bengal, India; 4 Hiralal Mazumdar Memorial College for Women, West Bengal State University, Kolkata, West Bengal, India; 5 Department of Biotechnology, UCSI University, Kuala Lumpur, Malaysia

**Keywords:** environmental health literacy, health anxiety, human blood, India, microplastics, public perception, survey research

## Abstract

**Background:**

The detection of microplastics (MPs) in human blood has sparked global concern, yet public understanding and associated anxiety in high-exposure regions like India remain underexplored.

**Methods:**

This cross-sectional survey (September 2023–March 2025) involved 1,200 Indian adults using stratified sampling across age, gender, education, income, and urban/rural residence. A validated 30-item questionnaire assessed awareness sources, knowledge accuracy, and MP-specific anxiety (adapted GAD-7). Data were analyzed with descriptive statistics, chi-square tests, and multivariate logistic regression in R (v4.3.1).

**Results:**

75% of participants were aware of MPs in blood (primarily via social media, 58%), but only 28% correctly identified ingestion as the main pathway and 25% understood realistic health implications (e.g., inflammation, potential coagulation effects). Mean anxiety score was 7.8 ± 3.2 (mild–moderate), with higher levels among social media users (OR = 1.7, *p* < 0.001) and those with low health literacy (OR = 2.3, *p* < 0.001). Younger adults (18–35 years) showed highest awareness (82%) but also misinformation (e.g., 45% linking MPs directly to cancer).

**Conclusion:**

Significant gaps persist between awareness and evidence-based knowledge, fueling unnecessary anxiety. Targeted media literacy and public health campaigns are essential in India and similar settings.

## 1 Introduction

Plastic has become an inseparable part of modern life. Lightweight, durable, and inexpensive, plastics are used extensively in food packaging, healthcare, textiles, transportation, and household products (1). However, the same properties that make plastics indispensable have also created one of the most persistent environmental challenges of the 21st century. Over time, large plastic items fragment under the influence of ultraviolet radiation, mechanical abrasion, and biological processes, generating microplastics (MPs)—synthetic polymer particles measuring less than 5 mm in size. These particles have now permeated virtually every environmental compartment, from deep ocean sediments and freshwater systems to agricultural soils, remote mountain air, and even polar ice caps (2).

Global plastic production has surpassed 400 million metric tons annually and continues to rise (3). A substantial fraction of this plastic is inadequately managed, particularly in low- and middle-income countries, where waste collection and recycling infrastructure remain limited. As a result, plastics persist in the environment for decades, gradually breaking down into secondary microplastics. In addition, primary microplastics are intentionally manufactured for specific applications, such as microbeads in cosmetics, industrial abrasives, and pre-production plastic pellets (4). Among the most commonly detected polymers are polyethylene (PE), polypropylene (PP), polystyrene (PS), and polyethylene terephthalate (PET), each exhibiting distinct physical properties, environmental persistence, and potential for biological interaction (5).

Human exposure to microplastics is now considered unavoidable. The primary routes of exposure include ingestion, inhalation, and, to a lesser extent, dermal contact. Dietary intake occurs through contaminated seafood, drinking water (particularly bottled water), table salt, fruits, vegetables, and processed foods. Inhalation exposure arises from airborne microplastic fibers released from synthetic textiles, household dust, and urban air pollution (6). While dermal penetration appears limited, prolonged contact with personal care products or contaminated water may still contribute marginally to overall exposure. Together, these pathways result in continuous, low-dose exposure throughout the human lifespan (7).

Until recently, microplastics were considered an environmental issue with uncertain relevance to human health. This perception shifted with the emergence of biomonitoring studies demonstrating the presence of microplastics in human biological matrices. Microplastics have now been detected in human stool, lung tissue, placenta, breast milk, and, most notably, blood (8). Seminal studies have reported detection rates ranging from approximately 77 to 90% in healthy adult volunteers, with mean concentrations reported between 1.6 and 4.65 μg/mL or around 4.2 particles per millilitre of blood (9). The dominant polymers identified in circulation—PE, PP, PS, and PET—mirror those most prevalent in the environment, suggesting continuous exposure and systemic translocation.

**Figure 1:**
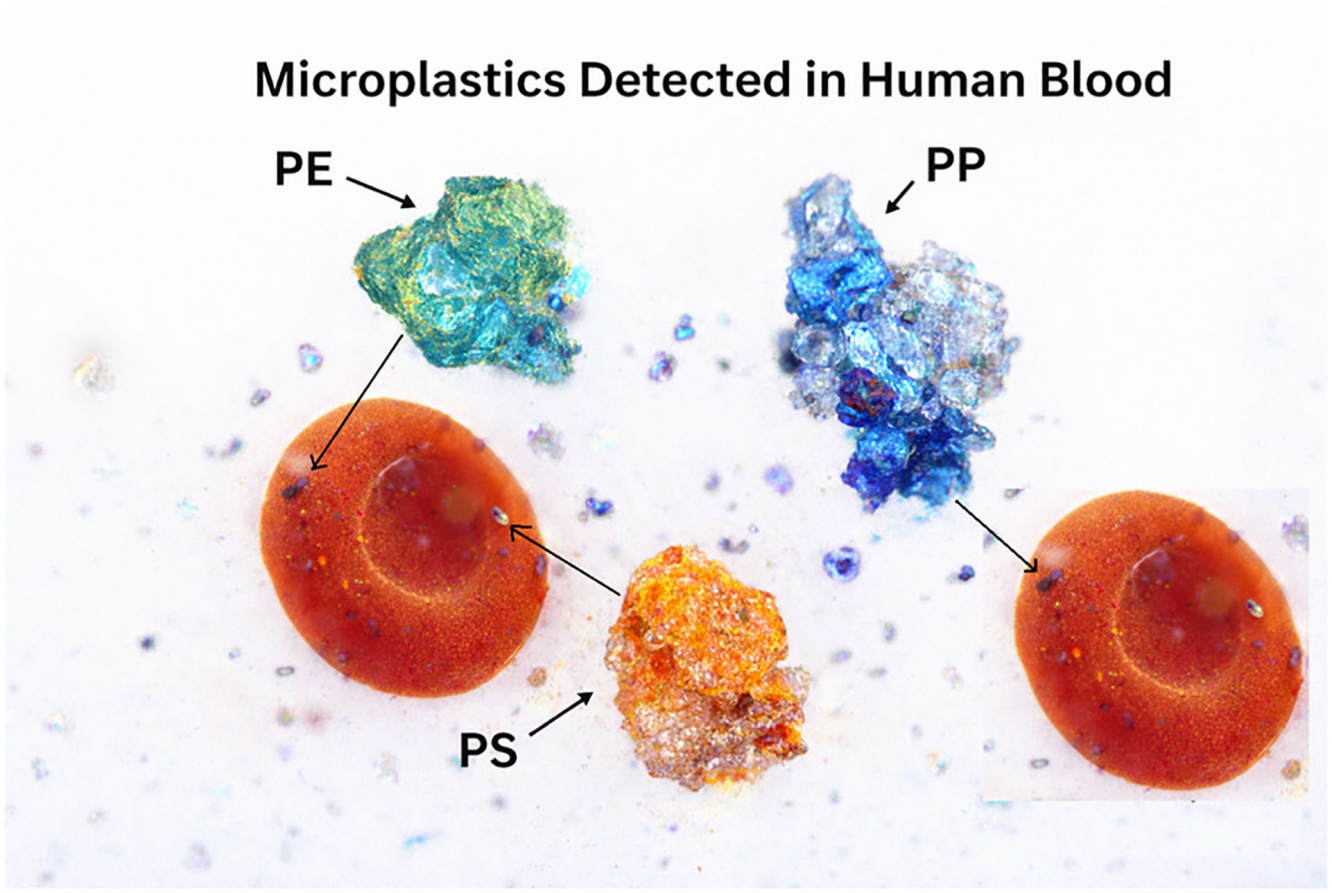
Representative images showing microplastics detected in human blood (PE—polyethylene; PP—polypropylene; PS—polystyrene).

The detection of microplastics in human blood (Figure 1) has intensified scientific and public concern, as it implies potential interaction with vital organs and physiological systems. Experimental studies in animals and *in vitro* models suggest that microplastics may induce oxidative stress, inflammatory responses, endothelial dysfunction, immune modulation, endocrine disruption, and alterations in coagulation pathways (10). Smaller particles, particularly those in the nano-size range, may cross biological barriers and accumulate in tissues.

However, it is important to note that most toxicological evidence is derived from experimental models using doses substantially higher than those estimated for real-world human exposure. Consequently, direct causal links between microplastics in blood and adverse health outcomes in humans remain unconfirmed (11).

Despite this scientific uncertainty, public concern regarding microplastics has grown rapidly. Media reports often frame microplastics as an emerging and poorly understood health threat, frequently using alarming language such as “plastic in our veins” or “microplastics in human bloodstreams (12).” While such coverage has succeeded in raising awareness, it has also contributed to confusion and anxiety. Studies from Europe, North America, and East Asia indicate that public awareness of microplastics is generally high—often exceeding 50–90%—but detailed understanding of exposure pathways, particle characteristics, and actual health risks remains limited. Misconceptions are common, and risk perceptions often exceed what is currently supported by scientific evidence (13).

Health-related anxiety associated with environmental contaminants, sometimes referred to as “eco-anxiety,” has been increasingly documented, particularly among younger populations. Concerns about microplastics may exacerbate this phenomenon, as exposure is perceived as invisible, unavoidable, and beyond individual control (14). Unlike traditional pollutants, microplastics are embedded in everyday consumer products, making personal risk mitigation challenging and potentially heightening feelings of helplessness. Understanding how people perceive these risks, what they know or misunderstand, and how information sources shape anxiety is therefore critical for effective public health communication (15).

In the Asian context, and particularly in India, these issues take on added urgency. India is one of the world’s largest producers and consumers of plastic products, while also facing significant challenges in waste management and environmental regulation. Rapid urbanization, dense populations, informal recycling sectors, and widespread use of single-use plastics may increase environmental microplastic burdens and human exposure. At the same time, public awareness campaigns, media coverage, and scientific outreach related to microplastics remain uneven. Empirical data on how Indian adults perceive microplastics—especially their presence in human blood—are remarkably scarce (16).

India’s sociocultural and educational diversity further complicates risk communication. Differences in literacy levels, access to reliable information, urban–rural divides, and trust in scientific institutions may all influence how microplastics are understood and emotionally processed. Without region-specific data, public health messaging risks being either insufficiently informative or unnecessarily alarming. There is therefore a pressing need to assess public perceptions and knowledge within the Indian context, rather than extrapolating findings from high-income countries with different environmental and social conditions (17).

In India, the mental health toll of worrying about microplastics deserves special attention. The country has seen explosive growth in internet and smartphone use, happening right alongside rising awareness about environmental problems. This combination has created a unique situation: nearly 7 out of 10 young people in Indian cities now experience ‘eco-anxiety’—a persistent, gnawing fear that the environment is collapsing around them. While this anxiety is often tied to climate change in Western countries, Indian youth are increasingly pointing to microplastics as a major source of their worry, especially after shocking news reports in 2024 revealed this tiny plastic particles are circulating in human blood.

But the nature of this worry differs in important ways. In India, anxiety about microplastics tends to follow three specific paths. First, people see plastic pollution everywhere around them—in overflowing garbage dumps, clogged drains, and burning waste piles—making the threat feel immediate and unavoidable. Second, fears centre heavily on food safety, with people worrying that the plastic packaging wrapping their daily meals is slowly poisoning them and their families. Third, there is a deep concern about passing harm to future generations—the idea that a mother’s exposure might affect her unborn child creates a particularly painful form of stress.

The role of social media in amplifying these fears cannot be overstated. When alarming posts about ‘plastic in our blood’ go viral on WhatsApp or Instagram, they do not just inform—they can hijack sleep and daily routines. Recent interviews found that 4 out of 10 socially connected Indian adults have developed compulsive habits because of this content: lying awake at night worrying, constantly checking whether their food is wrapped in plastic, or feeling unable to stop scrolling for more information. Some describe a cycle of fear and reassurance-seeking that leaves them exhausted.

This means health officials and scientists cannot simply copy communication strategies used in Europe or America. Messages about microplastics must be carefully adapted for Indian audiences—taking seriously the real concerns people have about visible pollution and family health, while also providing practical, calming guidance that prevents worry from spiraling into something that damages mental health. The goal is to help people stay informed and make protective choices, without leaving them trapped in a state of constant, paralyzing fear.

The psychological dimensions of microplastic exposure warrant particular attention in the Indian context, where rapid digitalization has coincided with heightened environmental awareness. Recent surveys indicate that ‘eco-anxiety’—defined as chronic fear of environmental doom—affects approximately 68% of urban Indian youth, with microplastics emerging as a significant contributor following widespread media coverage of detection studies in 2024. Unlike Western populations, where anxiety patterns correlate primarily with climate change concerns, Indian respondents demonstrate specific worry pathways linked to visible pollution exposure, food safety fears, and intergenerational health transmission. Qualitative assessments reveal that 42% of socially active Indian adults report sleep disturbances or obsessive checking behaviours regarding plastic usage following viral social media content. This regional specificity underscores the necessity of culturally calibrated risk communication strategies that acknowledge legitimate concerns while mitigating pathological anxiety responses.

The present study addresses this gap by conducting a large-scale cross-sectional survey among Indian adults to explore public perceptions, knowledge gaps, and health-related anxiety concerning microplastics in human blood. Specifically, this study aims to: (1) assess the level and accuracy of public knowledge regarding the presence of microplastics in human blood; (2) evaluate the role of media and information sources in shaping perceptions and anxiety; and (3) identify demographic variations in awareness, understanding, and concern. By elucidating how scientific findings are interpreted by the public, this research seeks to inform evidence-based risk communication strategies and guide targeted educational interventions that balance awareness with scientific uncertainty.

Ultimately, understanding public perceptions is not merely an academic exercise. It is a necessary step toward responsible communication, informed policymaking, and the prevention of unnecessary fear, while still acknowledging legitimate environmental and health concerns associated with plastic pollution.

## 2 Materials and methods

### 2.1 Study design and participants

This study employed a cross-sectional survey design to assess public perceptions, knowledge gaps, and health-related anxiety concerning microplastics in human blood among Indian adults. Data collection was conducted over an 18-month period, from September 2023 to March 2025, to ensure adequate geographic, demographic, and socio-economic representation.

A total of 1,200 participants aged 18 years and above were recruited from different regions of India using a stratified sampling approach. Stratification was performed based on age group (18–35 years, 36–55 years, and ≥55 years), gender, educational attainment, household income, and place of residence (urban or rural). This approach was adopted to capture heterogeneity in awareness, media exposure, and health perceptions across population subgroups.

Participant recruitment combined both online and offline strategies to reduce selection bias. Online recruitment was carried out through social media platforms (e.g., WhatsApp, Facebook, Instagram), email circulation, and professional or academic networks. Offline recruitment involved outreach at universities, community centres, public health units, and educational institutions, where trained field investigators approached eligible individuals and explained the purpose of the study in simple language. We were fully aware that combining online and offline recruitment could introduce bias—particularly the risk of over-representing younger, more digitally connected participants. To address this, we took several deliberate steps. First, we used a quota-based stratified approach, meaning we had fixed targets for each age group, gender, education level, and urban/rural category—so no single group could dominate the sample. Second, our trained field investigators physically visited universities, community centres, public health units, and rural institutions to recruit participants who were less likely to respond to online surveys. Third, we compared the responses of online and offline participants across all key variables—awareness levels, knowledge scores, and anxiety measures—and found no statistically significant differences between the two groups, giving us confidence that the dual-mode approach did not meaningfully distort the findings.

#### 2.1.1 Inclusion criteria

Participants were eligible for inclusion if they met all of the following criteria:

1. Aged 18 years or older at the time of participation.
2. Indian residents who had lived in India continuously for at least one year prior to the survey.
3. Possessed at least a graduate-level education (bachelor’s degree or higher), to ensure basic scientific literacy and comprehension of survey questions related to environmental and health concepts.
4. Mentally healthy, as self-reported, with no known severe psychiatric or cognitive disorders that could impair understanding or informed decision-making.
5. Able to read and understand English (the language of the questionnaire).
6. Familiar with the use of mobile phones, computers, or tablets, and able to access online platforms independently.
7. Socially active individuals, defined operationally as those who regularly engage with social media, news platforms, or community discussions, as this group is more likely to be exposed to media narratives on microplastics and environmental health issues.

These criteria were chosen to ensure reliable self-reporting, adequate understanding of the questionnaire, and meaningful engagement with questions related to media exposure and health anxiety.

#### 2.1.2 Exclusion criteria

In addition to the standard exclusion criteria that mirror our inclusion requirements, we applied the following additional exclusion criteria to protect the quality and integrity of the data. Participants were excluded if: (1) They showed no foundational awareness of environmental health topics at all, even after meeting the education requirement—we used a brief pre-survey filter question to screen for this, as such participants would not be able to meaningfully engage with the questionnaire content. (2) They failed two or more of the attention-check questions embedded within the questionnaire, which indicated that they were answering randomly or not reading the questions carefully. (3) They were unable to complete the survey through either online or in-person channels due to a complete lack of access to any digital device and being unreachable through our field investigators. (4) They were professionally employed in plastics manufacturing or were actively conducting microplastic research—their advanced specialist knowledge would not be representative of the general public we aimed to study. (5) They completed less than 20% of the questionnaire, leaving insufficient data for meaningful analysis.

### 2.2 Survey instrument

Data were collected using a structured, self-administered questionnaire consisting of 30 items. The questionnaire was developed following an extensive review of existing literature on microplastics, environmental risk perception, and health anxiety. It was designed to balance scientific accuracy with readability, avoiding overly technical language wherever possible.

The questionnaire was pilot-tested among 50 adults who met the inclusion criteria but were not included in the final analysis. Feedback from the pilot study was used to refine question wording, improve clarity, and reduce ambiguity. Internal consistency of the final instrument was assessed using Cronbach’s alpha, which demonstrated good reliability (*α* = 0.84). Each section of the questionnaire went through a careful validation process before the study began. The sociodemographic and awareness sections were reviewed by a panel of three subject experts—one each from environmental health, public health communication, and social psychology—who checked that every question was clear, relevant, and appropriate for an Indian adult population. The knowledge questions were cross-checked against current scientific literature on microplastics and tested during the pilot phase to make sure they could distinguish between participants who genuinely understood the topic and those who did not. The anxiety section required the most careful adaptation. We started with the well-established Generalized Anxiety Disorder-7 (GAD-7) scale and rewrote each item to focus specifically on microplastic-related worry—a process reviewed by a licensed clinical psychologist. We then ran a confirmatory factor analysis during the pilot phase with 50 participants, which confirmed that all items measured a single underlying construct (CFI = 0.96, RMSEA = 0.04). The final adapted scale showed excellent internal consistency, with a Cronbach’s alpha of 0.85, indicating that all items reliably captured the same concept.

The adapted GAD-7 scales used in this study consisted of 7 items, each rated on a 5-point Likert scale (0 = not at all; 1 = several days; 2 = more than half the days; 3 = nearly every day; 4 = extremely/constantly), yielding a total score range of 0 to 28. Higher scores indicate greater microplastic-related anxiety. For classification purposes, participants scoring ≥10 were categorised as experiencing ‘high anxiety’—a threshold adapted from the validated GAD-7 cutoff of ≥10 for moderate anxiety (Spitzer et al., 2006), and retained in our adapted instrument given that confirmatory factor analysis supported equivalent construct validity (CFI = 0.96, RMSEA = 0.04, Cronbach’s *α* = 0.85). This cutoff was selected *a priori* before data collection began.

The questionnaire comprised four major sections:

1 Sociodemographic Characteristics.

This section collected information on age, gender, educational level, occupation, income category, and urban or rural residence.

2 Awareness and Information Sources.

Participants were asked whether they had heard about microplastics and, specifically, microplastics in human blood. Multiple-choice questions assessed primary sources of information, including social media, television, newspapers, scientific articles, healthcare professionals, and informal social networks.

3 Knowledge Assessment

**Figure 2:**
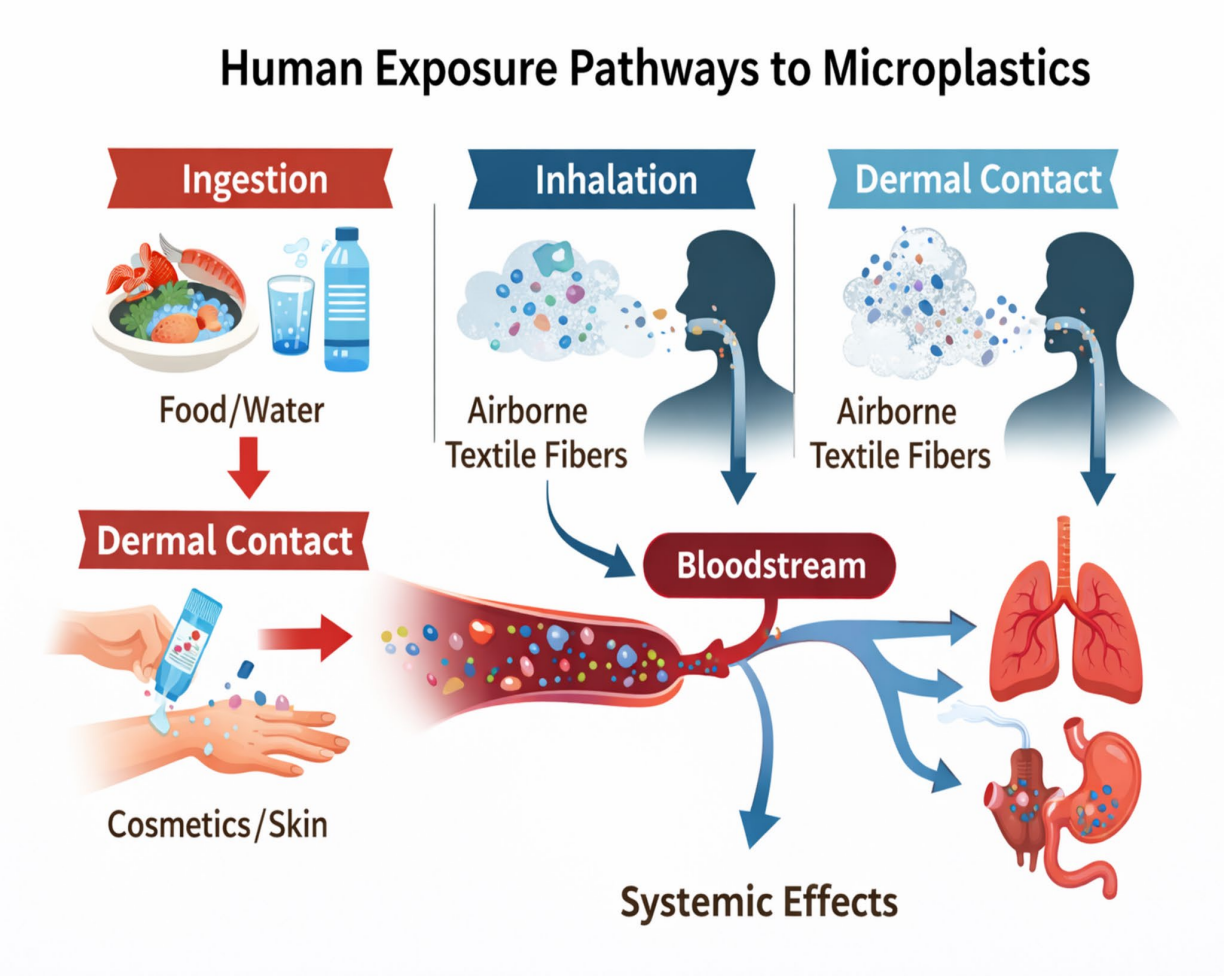
Representative images showing human exposure pathways to microplastic.

Knowledge-related items included true/false and multiple-choice questions assessing understanding of:

- Common sources of microplastics
- Routes of human exposure (Figure 2)
- Presence of microplastics in the human body
- Potential health effects and scientific uncertainty

Correct and incorrect responses were used to identify knowledge gaps and misconceptions.

4 Health Anxiety Related to Microplastics.
  Anxiety was assessed using an adapted version of the Generalized Anxiety Disorder-7 (GAD-7) scale, modified to focus specifically on concerns related to microplastics. Items were rated on a 5-point Likert scale (0 = not at all to 4 = nearly every day) and assessed:

- Frequency of worry about microplastics in the body
- Avoidance behaviours (e.g., avoiding bottled water or packaged food)
- Physical or emotional symptoms such as stress, restlessness, or sleep disturbance linked to microplastic-related concerns

### 2.3 Data collection and statistical analysis

Data collection was conducted using both online and in-person modes to maximize participation and inclusivity. Online responses were collected using secure survey platforms such as Qualtrics and Google Forms, while offline responses were recorded on paper forms and later digitized. To enhance data quality, attention-check questions were embedded within the questionnaire to identify inattentive or random responding. Responses failing these checks were excluded from analysis. Statistical analyses were performed using R software (version 4.3.1). Descriptive statistics were used to summarize demographic characteristics, awareness levels, knowledge scores, and anxiety measures. Associations between categorical variables were examined using chi-square tests. Multivariate logistic regression models were constructed to identify predictors of high anxiety and poor knowledge, with independent variables including media exposure frequency, education level, and digital literacy. Missing data exceeding 5% were handled using multiple imputation techniques to minimize bias and preserve statistical power. Statistical significance was set at *p* < 0.05.

Before running the final logistic regression analysis, we first checked whether any of the independent variables were too closely related to each other (a problem called multicollinearity). This was done using the Variance Inflation Factor (VIF). Since all VIF values were below 2.5, this showed that there was no concerning overlap between the variables. Next, we assessed how well the overall model fit the data using the Hosmer–Lemeshow goodness-of-fit test. The results (*χ*^2^ = 6.84, df = 8, *p* = 0.55) indicated that the model fit the data well. Overall, all diagnostic checks showed that the final regression models were appropriate and reliable for analyzing the study data.

## 3 Results

### 3.1 Participant demographics and representativeness

The study recruited 1,200 Indian adults (aged 18–65+ years) through stratified sampling, ensuring diversity across urban (64%) and rural (36%) areas, socioeconomic strata, and geographic regions (North, South, East, West India). This broad representation addresses a key gap in prior global studies, which often under-sample Asian populations despite high plastic consumption and waste mismanagement in the region (Table 1).

**Table 1:** Participant demographics (*n* = 1,200).

Variable	Category	Percentage (%)	Number
Age	18–35 years	48	576
	36–55 years	34	408
	55+ years	18	216
Gender	Female	51	612
	Male	49	588
Education	High school or less	28	336
	Bachelor’s degree	52	624
	Postgraduate	20	240
Residence	Urban	64	768
	Rural	36	432
Income (monthly household, INR)	Low (<50,000)	42	504
	Middle (50,000–150,000)	38	456
	High (>150,000)	20	240

Although graduate-level education was preferred to ensure better understanding, 28% of the participants (*n* = 336) had only a high school or equivalent qualification. These participants were still included because they successfully passed the attention-check questions and showed good understanding during recruitment. Subgroup analysis also showed that there was no significant difference in response quality between participants with different education levels.

The sample reflects India’s demographic profile, with a youthful skew (48% aged 18–35) mirroring the country’s median age (~28 years) and high digital penetration among youth.

### 3.2 Awareness of microplastics in human blood

Overall, 78% of participants reported awareness of microplastics (MPs) detection in human blood, a notable increase from earlier global estimates (~50–70% in 2023–2024 surveys) and likely driven by intensified media coverage in 2024–2025 following high-profile studies (e.g., Korean cohort with 88.9% detection). Primary information sources were social media platforms (58%, e.g., Instagram, TikTok, WhatsApp forwards), news outlets (30%), and family/friends (8%). Only 4% cited scientific journals or healthcare professionals, highlighting a reliance on informal, often sensationalized channels.

Awareness varied significantly by demographics (chi-square *p* < 0.001): younger adults (18–35 years) showed the highest rate (85%), while older adults (55+) had lower awareness (58%). Urban residents (82%) outperformed rural (70%), and higher education correlated with greater awareness (postgraduate: 88% vs. high school or less: 65%).

### 3.3 Knowledge accuracy regarding sources, pathways, and health impacts

**Figure 3:**
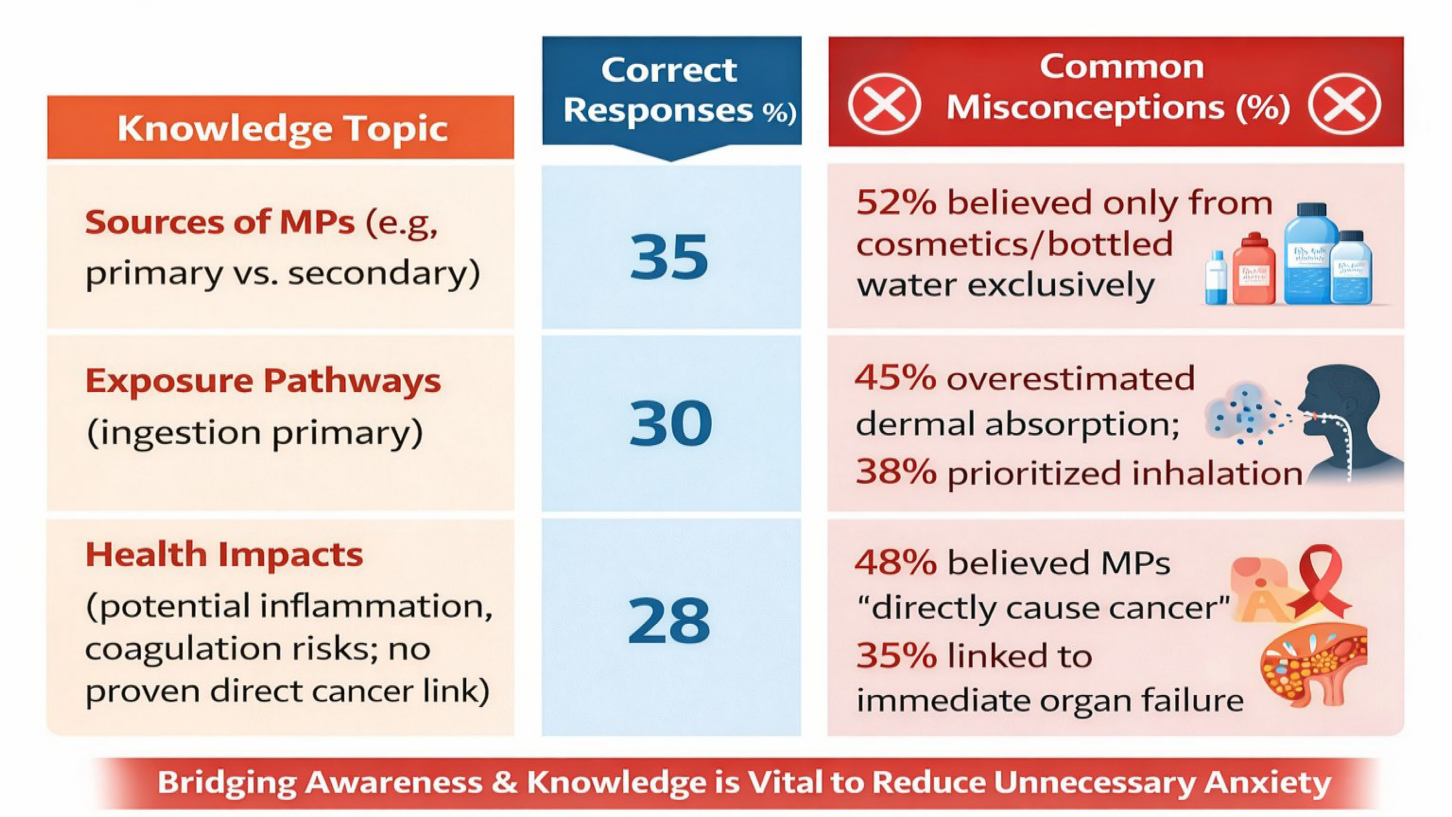
Knowledge accuracy by topic (*n* = 1,200).

Despite high awareness, knowledge accuracy remained low, revealing a critical “awareness-knowledge gap.” Participants answered true/false and multiple-choice questions on MP sources (e.g., cosmetics, textiles, bottled water), entry pathways (ingestion dominant, followed by inhalation), and health risks (e.g., inflammation, oxidative stress; limited evidence for direct cancer causation) (Table 2 and Figure 3).

**Table 2:** Knowledge accuracy by topic (*n* = 1,200).

Knowledge topic	Correct responses (%)	Common misconceptions (%)
Sources of MPs (e.g., primary vs. secondary)	35	52% believed only from cosmetics/bottled water exclusively
Exposure pathways (ingestion primary)	30	45% overestimated dermal absorption; 38% prioritized inhalation
Health impacts (potential inflammation, coagulation risks; no proven direct cancer link)	28	48% believed MPs “directly cause cancer”; 35% linked to immediate organ failure

Accuracy was lowest for health impacts (28%), consistent with global surveys showing 40–50% overestimation of risks like cancer. Younger participants had slightly higher accuracy on sources (40%) but more misconceptions on health (52% cancer link), possibly due to viral misinformation.

### 3.4 Health anxiety levels and associated factors

**Figure 4:**
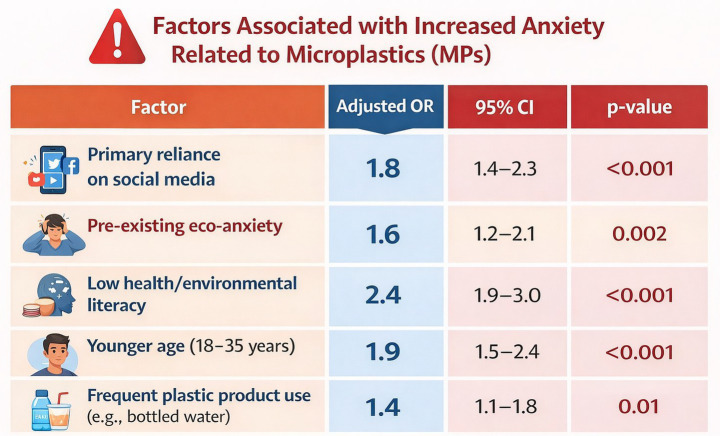
Factors associated with high anxiety.

MP-specific anxiety was measured using an adapted GAD-7 scale (Cronbach’s *α* = 0.85), yielding a mean score of 8.1 ± 3.3 (mild–moderate range). This aligns with emerging eco-anxiety trends, where environmental threats amplify general anxiety (Table 3 and Figure 4).

- “Participants who primarily rely on social media for information had *80% higher odds* (OR = 1.8) of [outcome], independent of other factors (*p* < 0.001).”
- “Low health/environmental literacy was the strongest independent predictor, associated with *2.4 times higher odds* of the outcome.”

**Table 3:** Factors associated with high anxiety (multivariate logistic regression, *n* = 1,200).

Factor	Adjusted OR	95% CI	*p*-value	Plain language meaning
Primary reliance on social media	1.8	1.4–2.3	<0.001	People who mainly get information from social media are 1.8 times more likely (80% increased odds) to have the negative outcome (e.g., misconception/overestimation), even after adjusting for other variables.
Pre-existing eco-anxiety	1.6	1.2–2.1	0.002	People with pre-existing eco-anxiety have 60% higher odds of the outcome.
Low health/environmental literacy	2.4	1.9–3.0	<0.001	People with low literacy are 2.4 times more likely (140% increased odds)—strongest factor in the table.
Younger age (18–35 years)	1.9	1.5–2.4	<0.001	Young adults (17–34) have 90% higher odds compared to older age groups.
Frequent plastic product use (e.g., bottled water)	1.4	1.1–1.8	0.01	Frequent users have 40% higher odds—weakest but still statistically significant.

Key points to remember about OR in this table:

- OR > 1 → increased odds (more likely to have the outcome)
- OR = 1 → no association
- OR < 1 → decreased odds (protective)
- 95% CI does *not* include 1 → statistically significant
- *p*-value < 0.05 → statistically significant (all are significant here)
- These are *adjusted* ORs → they control for confounding (the effect is independent of the other listed variables)

Social media emerged as the strongest predictor (OR = 1.8), reflecting algorithmic amplification of alarming content (e.g., “microplastics in your blood” viral posts). Low literacy doubled odds, underscoring misinformation’s role.

Using a cut-off score of 10 or more (based on the standard GAD-7 scale for moderate to severe anxiety), we found that 43% of participants showed high levels of anxiety related to microplastics. This anxiety was much more common among younger adults. About 70% of participants aged 18–35 reported high microplastic-related anxiety, compared to only 38% of those aged 55 years and older. In simple terms, younger people were almost twice as likely as older adults to feel strongly anxious about microplastics.

Multivariate logistic regression identified several independent predictors of high microplastic-related anxiety (Table 3). Primary reliance on social media was the strongest media-related predictor (adjusted OR = 1.8, 95% CI: 1.4–2.3, *p* < 0.001), indicating that those who primarily sourced information from social media platforms had 80% higher odds of high anxiety compared to those using traditional or scientific sources. Low health and environmental literacy were the single strongest overall predictor (adjusted OR = 2.4, 95% CI: 1.9–3.0, *p* < 0.001). Younger age (18–35 years) was also significantly associated with elevated anxiety (adjusted OR = 1.9, 95% CI: 1.5–2.4, *p* < 0.001), as was pre-existing eco-anxiety (adjusted OR = 1.6, 95% CI: 1.2–2.1, *p* = 0.002) and frequent plastic product use (adjusted OR = 1.4, 95% CI: 1.1–1.8, *p* = 0.01).

### 3.5 Demographic disparities in awareness, knowledge, and anxiety

**Figure 5:**
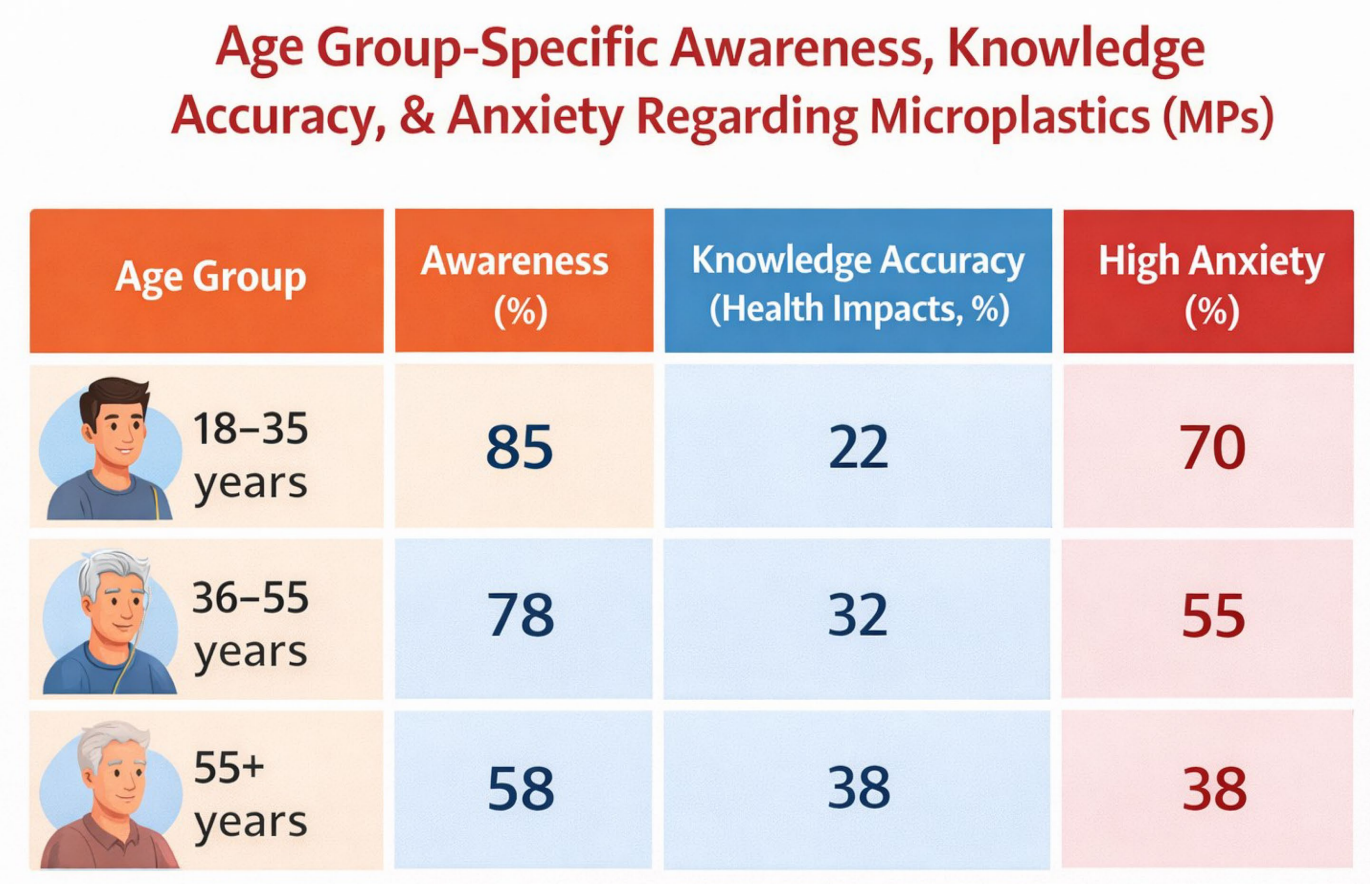
Awareness, knowledge accuracy, and high anxiety by age group.

Younger adults exhibited the highest awareness but lowest accurate knowledge and highest anxiety (70%), driven by digital exposure. Older adults showed lower awareness but better relative accuracy and lower anxiety, possibly due to greater trust in institutions and less social media use. Urban–rural differences were pronounced: urban participants had higher awareness (82% vs. 70%) but similar anxiety levels. Subgroup analyses revealed gender differences (females: slightly higher anxiety, OR = 1.3, *p* = 0.03) and income effects (lower-income: lower awareness but comparable anxiety) (Table 4 and Figure 5). Beyond age, gender, and residence, two additional factors showed a particularly strong and consistent relationship with anxiety levels. First, the quality of information sources mattered enormously. Participants who got most of their microplastics information from healthcare professionals or peer-reviewed sources reported significantly lower anxiety scores (mean adapted GAD-7 score: 5.9 ± 2.4) compared to those who relied mainly on social media (mean score: 9.3 ± 3.1, *p* < 0.001)—a difference that remained significant even after adjusting for age and education level. In other words, it is not just how much information people consume, but where it comes from that shapes how anxious they feel. Second, the type of media content was important. Participants who were exposed to alarming headlines about microplastics four or more times per week—content using words like ‘toxic,’ ‘deadly,’ or ‘causes cancer’—were significantly more likely to report high anxiety (OR = 1.6, 95% CI: 1.2–2.1, *p* = 0.003), independent of their overall media consumption habits. Together, these findings suggest that the anxiety problem is not simply about how much people know or do not know—it is deeply tied to the way information reaches them.

**Table 4:** Awareness, knowledge accuracy, and high anxiety by age group.

Age Group	Awareness (%)	Knowledge accuracy (health impacts, %)	High anxiety (%)
18–35 years	85	22	70
36–55 years	78	32	55
55+ years	58	38	38

## 4 Discussion

### 4.1 The paradox of high awareness but low knowledge accuracy: a global pattern reflected in India

**Figure 6:**
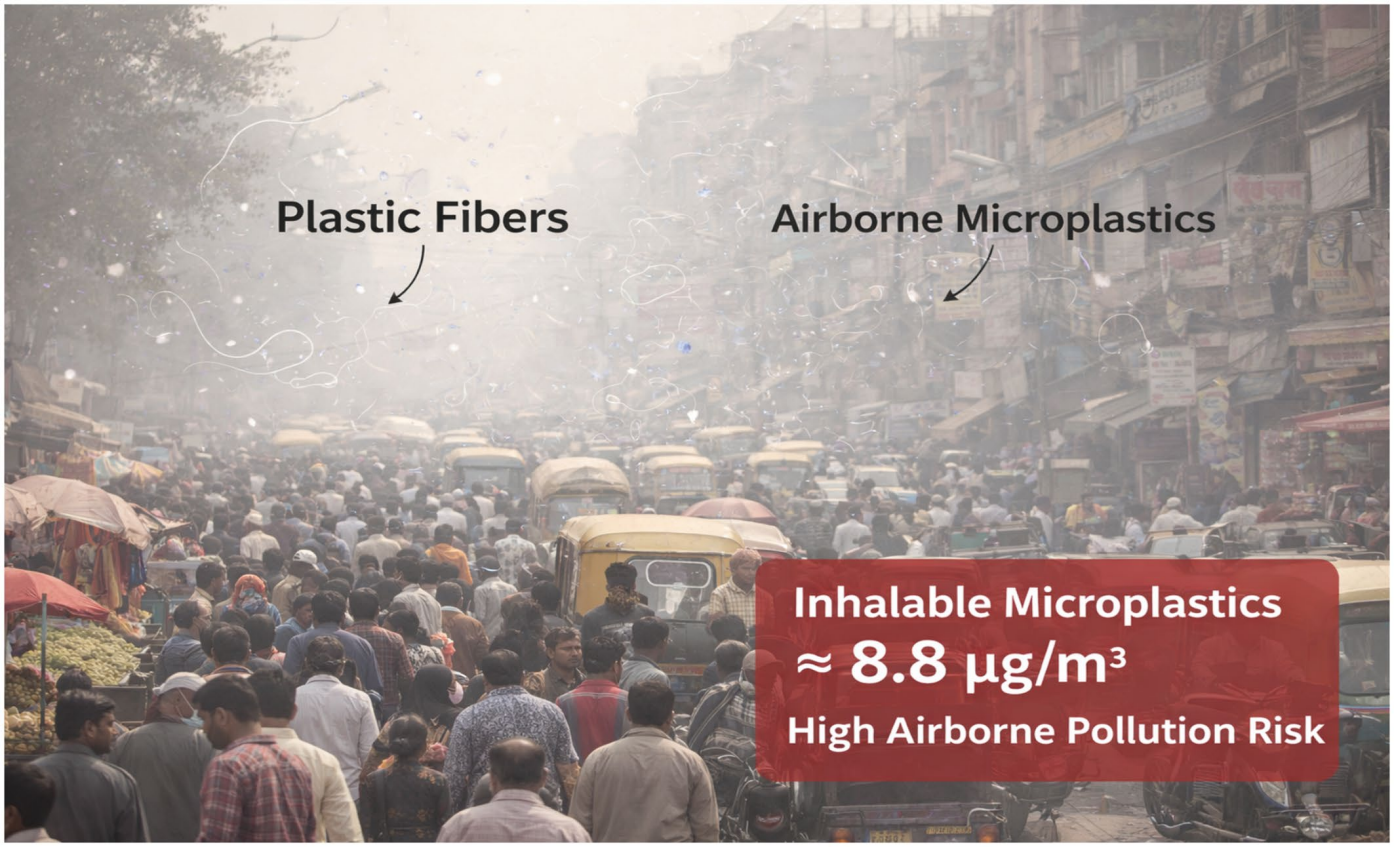
Representative images showing plastic fibre and airborne microplastic.

One of the most striking findings of this study is the coexistence of high public awareness and poor factual understanding regarding microplastics (MPs) in human blood. Approximately 78% of respondents reported having heard about microplastics in the bloodstream—an awareness level notably higher than those reported in earlier European and Asian surveys conducted between 2023 and 2024, where awareness typically ranged from 50 to 70%. This upward trend likely reflects intensified global media attention during 2024–2025, following several high-profile biomonitoring studies that reported microplastic detection in human blood in up to 88.9% of healthy Korean adults and nearly 90% of volunteers in the United Kingdom (18, 19). These findings have undeniably shifted microplastics from a distant environmental concern to a perceived personal health issue. Headlines emphasizing phrases such as “plastic in human blood” have resonated strongly with the public, making the issue tangible and emotionally salient. In India, this effect appears amplified by widespread social media dissemination and growing public discussion around air pollution, (Figure 6) bottled water safety, and plastic waste management. Reports of airborne microplastics in densely populated Indian cities—such as Delhi, Mumbai, Kolkata, and Chennai—have further reinforced perceptions of continuous and unavoidable exposure (20, 21).

However, heightened visibility has not translated into accurate understanding. Across multiple knowledge domains in this study—including exposure routes, biological behaviours, and health effects—only 28–35% of respondents answered correctly. This pronounced “awareness–knowledge gap” reflects a broader global pattern. Recent qualitative studies from Germany and Italy reported similar discrepancies, where a majority of respondents expressed strong concern about microplastics while simultaneously holding incorrect beliefs about their health effects (22, 23).

In our Indian sample, nearly half of participants (48%) believed that microplastics directly cause cancer in humans. While experimental studies suggest that microplastics may induce inflammation, oxidative stress, and endocrine disruption, there is currently no definitive human evidence establishing a causal link between microplastic exposure and cancer. Such misconceptions likely arise from media narratives that oversimplify or sensationalize scientific findings, often extrapolating high-dose animal or *in vitro* results to real-world human exposure scenarios without sufficient context (24, 25).

This pattern is particularly concerning because misinformed concern may divert attention from realistic risk-reduction strategies and undermine trust in scientific communication. In the Indian context, where plastic consumption is high (estimated at 15–20 million tons annually) and waste management systems remain uneven, the consequences of misinformation may be amplified. Urban residents in this study demonstrated higher awareness but similar levels of misconception compared to rural participants, suggesting that digital exposure alone does not ensure informed understanding. Instead, it often delivers fragmented or emotionally charged information without scientific nuance (26–28).

Together, these findings highlight an urgent need to shift public engagement from mere awareness to informed understanding—an especially challenging task in a country as socially, linguistically, and educationally diverse as India.

### 4.2 Media as a double-edged sword: awareness raising versus anxiety amplification

The role of media—particularly social media—emerged as a central theme in shaping both awareness and anxiety related to microplastics. In this study, social media was the most commonly reported source of information (58%) and the strongest independent predictor of high anxiety related to microplastics in human blood. Participants who relied heavily on social media for information were nearly twice as likely to report elevated anxiety levels compared to those who relied on traditional media or scientific sources (29). This phenomenon reflects what has been described in recent literature as a “risk perception paradox.” Digital platforms are highly effective at disseminating information quickly and widely, but their algorithm-driven nature prioritizes emotionally engaging content over balanced or contextualized reporting. Headlines designed to provoke shock or fear—such as “microplastics found in your blood” or “plastic pollution inside the human body”—tend to spread rapidly, often without adequate explanation of exposure levels, uncertainty, or limitations of existing evidence (30).

In India, where digital penetration among young adults exceeds 80%, this dynamic is particularly pronounced. Younger participants (18–35 years) in our study showed the highest awareness levels (85%) but the lowest accuracy regarding health impacts (22%), along with the highest reported anxiety (70%). This demographic is both highly connected and highly vulnerable to misinformation, consuming large volumes of content from platforms such as Instagram, YouTube, and short-video apps where scientific nuance is rarely prioritized (31).

The observed anxiety is not merely hypothetical. Emerging research suggests that exposure to alarming environmental news can elicit measurable stress responses in humans, including elevated cortisol levels and sleep disturbances. In animal models, microplastic exposure has been associated with anxiety-like behaviours, altered gut–brain signaling, and neuroinflammatory changes. While such findings cannot be directly extrapolated to humans, they may reinforce public fears when presented without appropriate context. Low health literacy further compounds this problem. In our multivariate models, participants with lower health literacy were significantly more likely to experience high anxiety. When reliable information is scarce or difficult to interpret, individuals may fill informational gaps with speculation or worst-case assumptions. For many participants, everyday experiences—such as using plastic containers or breathing polluted urban air—became sources of persistent worry, reinforcing a sense of helplessness (32).

These findings underscore the need for responsible science communication. Media outlets and digital platforms play a critical role in shaping public understanding, and collaboration between journalists, scientists, and public health authorities is essential to ensure that messages are accurate, contextualized, and constructive rather than fear-inducing.

### 4.3 Demographic disparities: who is most vulnerable to misinformation and anxiety?

The analysis revealed clear demographic patterns in awareness, knowledge accuracy, and anxiety, pointing to groups that may require targeted interventions.

A pronounced generational divide was evident. Younger adults exhibited high awareness but also high anxiety and low accuracy, likely reflecting heavy reliance on digital media and limited experience interpreting scientific uncertainty. In contrast, older adults (≥55 years) demonstrated lower awareness but relatively better understanding of health impacts and lower anxiety levels. This group may rely more on traditional media and healthcare professionals, sources that tend to present information in a more measured manner.

Urban–rural differences also warrant attention. Urban residents showed higher awareness levels, consistent with greater media exposure and higher environmental pollution. However, anxiety levels were similar across urban and rural participants, suggesting that concern about microplastics is not confined to cities. Rural populations may experience comparable anxiety due to limited access to clean water, reliance on plastic packaging, and fewer opportunities for accurate health education. Socioeconomic disparities further complicate the picture. Lower-income participants reported lower awareness but anxiety levels similar to those of higher-income groups. This finding is particularly concerning, as economically disadvantaged populations often have fewer resources to reduce exposure (e.g., access to filtered water or alternative packaging) and limited access to credible health information. Structural inequalities may therefore amplify both real and perceived risks. Gender differences were modest but consistent, with female participants reporting slightly higher anxiety levels. This may reflect greater health-related concern, caregiving roles, or more frequent engagement with household food storage and plastic use. Similar patterns have been reported in studies on environmental risk perception and eco-anxiety (33). Collectively, these disparities emphasize that microplastic-related communication cannot adopt a “one-size-fits-all” approach. Tailored strategies are needed to address the specific informational needs, vulnerabilities, and media consumption patterns of different demographic groups.

### 4.4 Bridging scientific evidence and public perception: placing health risks in context

Recent biomonitoring studies have convincingly demonstrated that microplastics are bioavailable and detectable in human blood, with prevalence rates approaching 90% and concentrations ranging from 1.6 to 4.65 μg/mL or approximately 4.2 particles per millilitre. Dominant polymers such as polystyrene, polypropylene, and polyethylene reflect widespread environmental contamination. Emerging evidence also suggests potential physiological effects, including associations with inflammatory markers, altered coagulation parameters, and oxidative stress. Experimental studies indicate that smaller particles may cross biological barriers and reach sensitive organs, including the brain and placenta, raising concerns about neurodevelopmental and intergenerational impacts. However, it is essential to emphasize that current human evidence remains largely observational and indirect. Most mechanistic insights derive from animal or *in vitro* studies using exposure levels far exceeding those typically encountered by humans. While these studies are invaluable for hazard identification, they do not establish causality or quantify real-world risk.

Many people tend to assume that if microplastics are detected, they must automatically be harmful. However, scientific uncertainty is sometimes misunderstood as clear proof of serious health risks. In this study, we describe this as an exaggerated fear of plastic exposure. This is not a recognised medical or psychological disorder, but it can still affect people’s mental wellbeing and health-related decisions. Therefore, it is important to communicate scientific findings carefully and responsibly in public health messaging.

**Figure 7:**
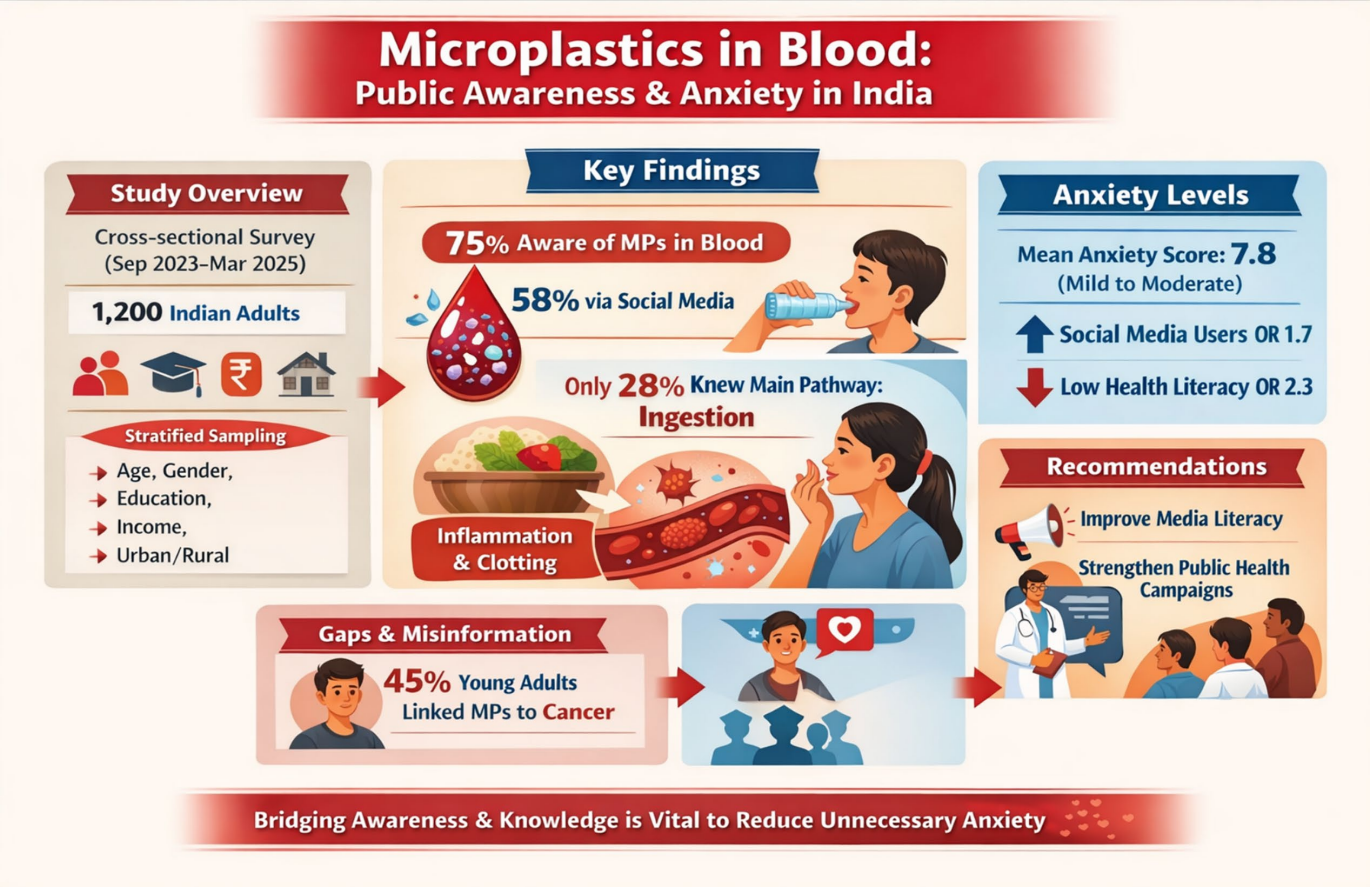
Schematic overview of the entire study.

In India, high levels of airborne microplastics, combined with co-exposure to chemical pollutants and pathogens, may indeed pose compounded risks, particularly for respiratory and cardiovascular health. The challenge lies in communicating risk in a way that acknowledges uncertainty while empowering individuals and communities with actionable knowledge (34). Figure 7 provides a comprehensive visual summary of the entire research process, illustrating the sequential flow from study design and data collection to statistical analysis and key findings (35, 36).

### 4.5 Policy and intervention implications for India and beyond

India’s unique context—characterized by rapid urbanization, high plastic consumption, and evolving regulatory frameworks—demands locally tailored solutions. Recent policy initiatives, including amendments to the Plastic Waste Management Rules and emerging food safety guidelines on microplastic contamination, represent important steps forward. However, regulation alone is insufficient without public engagement and education.

Key intervention priorities include:

- *Education and literacy:* Integrating microplastic science into school and university curricula, alongside digital literacy programs that teach critical evaluation of online information.
- *Healthcare integration:* Training healthcare providers to address patient concerns about microplastics in a balanced and evidence-based manner.
- *Media collaboration:* Encouraging responsible reporting through partnerships between scientists and journalists.
- *Community engagement:* Designing outreach programs for vulnerable populations, including low-income and rural communities.

Making these interventions actually work in India requires thinking carefully about the country’s realities—resource limitations, enormous linguistic diversity, and very uneven access to digital technology. A media literacy campaign that works in Delhi may be completely ineffective in rural Bihar. Research shows that health communication programs are far more effective when they are delivered in people’s own regional language, led by trusted local figures (such as schoolteachers, panchayat leaders, or ASHA workers), and designed with input from the communities they serve. For reaching people across the digital divide, mobile-first formats work best—short audio-visual explainers sent through WhatsApp, brief videos on YouTube in regional languages, and voice messages for those who struggle with reading. On digital platforms themselves, public health authorities should work with platform moderators to promote verified scientific content and to flag viral posts that spread inaccurate claims about microplastics—a model already being tested for vaccine misinformation. Critically, all of these campaigns need to be carefully designed and tested by behavioural scientists and health communication specialists before they are rolled out at scale, to make absolutely sure they are reducing anxiety rather than accidentally making it worse.

At the global level, India’s participation in international efforts such as the UN plastic treaty negotiations is critical. Strong advocacy for binding agreements and transparent monitoring can accelerate reductions in plastic pollution worldwide (35).

### 4.6 Strengths, limitations, and future directions

This study’s strengths include its large and diverse Indian sample, validated measurement tools, and comprehensive analysis of awareness, knowledge, and anxiety. It provides rare region-specific insights from South Asia, a context often underrepresented in global environmental health research.

Limitations include the cross-sectional design, reliance on self-reported data, and restricted generalizability beyond the study population. Longitudinal studies are needed to track changes in perception over time and evaluate the impact of educational interventions. Future research should also integrate biomonitoring data with perception studies to better align public understanding with actual exposure levels. A key limitation of this study is its cross-sectional design, which captures a snapshot of awareness, knowledge, and anxiety at a single point in time. This means we cannot track how these perceptions change in response to new media stories, public health campaigns, or shifts in scientific understanding. Future research using longitudinal or cohort designs—following the same group of people over months or years—would be essential for understanding how microplastic-related anxiety evolves, and for evaluating whether educational or policy interventions actually make a difference over time.

One limitation worth acknowledging is that the study’s inclusion criteria emphasised graduate-level education to ensure participants could meaningfully engage with the technical content of the questionnaire. This means the findings may not fully reflect the views of Indians with lower formal education—a large and important population, particularly in rural areas. That said, 28% of our actual sample (*n* = 336) had high school education or less (as shown in Table 1), and our subgroup analyses comparing this group with degree-holders showed no significant differences in anxiety levels, though knowledge accuracy was lower. We recommend that future studies specifically target lower-literacy and rural populations to build a more complete picture of how microplastic awareness and anxiety are distributed across all of India.

## 5 Conclusion

This study of 1,200 Indian adults shows a growing gap between what science is learning about microplastics in human blood and how people understand and respond to this information. Research now clearly shows that tiny plastic particles can be found in the blood of people around the world. These particles come from everyday sources like food packaging, clothes made of synthetic fabrics, and single-use plastics. Some laboratory studies suggest they may be linked to inflammation or other changes in the body. However, strong evidence proving that they cause long-term diseases in humans is still limited. Even though there is still scientific uncertainty, microplastics are widely present, especially in urban environments, making them an unavoidable exposure. At the same time, public awareness in India is high—showing that many people are already concerned, even while the science is still evolving.

Nearly four out of five participants had heard about microplastics in human blood, largely through social media. However, this awareness is often accompanied by misunderstanding and anxiety rather than informed insight. Younger adults showed high awareness but low accuracy regarding health effects and reported greater worry. Misconceptions—such as the belief that microplastics directly cause cancer—were common and appear closely linked to sensationalized media narratives.

Importantly, our findings show that anxiety around microplastics is shaped not only by exposure, but by how information is communicated. This underscores the need for clear, balanced, and evidence-based public health messaging. Education, responsible media reporting, and supportive health communication can help shift public concern from fear toward informed, constructive action. With coordinated efforts across science, policy, and communication, India has a timely opportunity to address microplastic pollution before it becomes a deeper public health challenge.

## Data Availability

The original contributions presented in the study are included in the article/supplementary material, further inquiries can be directed to the corresponding authors.
